# Comparison of eye tracking, electrooculography and an auditory brain-computer interface for binary communication: a case study with a participant in the locked-in state

**DOI:** 10.1186/s12984-015-0071-z

**Published:** 2015-09-04

**Authors:** Ivo Käthner, Andrea Kübler, Sebastian Halder

**Affiliations:** Institute of Psychology, University of Würzburg, Marcusstr. 9-11, 97070 Würzburg, Germany; Department of Rehabilitation for Brain Functions, Research Institute of National Rehabilitation Center for Persons with Disabilities, 4-1 Namiki, Tokorozawa, Saitama 359-8555 Japan

## Abstract

**Background:**

In this study, we evaluated electrooculography (EOG), an eye tracker and an auditory brain-computer interface (BCI) as access methods to augmentative and alternative communication (AAC). The participant of the study has been in the locked-in state (LIS) for 6 years due to amyotrophic lateral sclerosis. He was able to communicate with slow residual eye movements, but had no means of partner independent communication. We discuss the usability of all tested access methods and the prospects of using BCIs as an assistive technology.

**Methods:**

Within four days, we tested whether EOG, eye tracking and a BCI would allow the participant in LIS to make simple selections. We optimized the parameters in an iterative procedure for all systems.

**Results:**

The participant was able to gain control over all three systems. Nonetheless, due to the level of proficiency previously achieved with his low-tech AAC method, he did not consider using any of the tested systems as an additional communication channel. However, he would consider using the BCI once control over his eye muscles would no longer be possible. He rated the ease of use of the BCI as the highest among the tested systems, because no precise eye movements were required; but also as the most tiring, due to the high level of attention needed to operate the BCI.

**Conclusions:**

In this case study, the partner based communication was possible due to the good care provided and the proficiency achieved by the interlocutors. To ease the transition from a low-tech AAC method to a BCI once control over all muscles is lost, it must be simple to operate. For persons, who rely on AAC and are affected by a progressive neuromuscular disease, we argue that a complementary approach, combining BCIs and standard assistive technology, can prove valuable to achieve partner independent communication and ease the transition to a purely BCI based approach. Finally, we provide further evidence for the importance of a user-centered approach in the design of new assistive devices.

**Electronic supplementary material:**

The online version of this article (doi:10.1186/s12984-015-0071-z) contains supplementary material, which is available to authorized users.

## Background

Neurodegenerative diseases such as amyotrophic lateral sclerosis (ALS) or lesions in the brainstem caused by stroke, traumatic or anoxic brain injury can lead to a locked-in syndrome (LIS). First coined by Plum and Posner in 1966 [1], the term describes a state in which persons are severely paralyzed (quadriparesis or quadriplegia), unable to speak (aphonia or severe dysphonia), but aware of their environment and show cognitive abilities on examination. Eye opening is sustained and the principle methods of communication are vertical or horizontal eye-movements or blinking (according to the criteria suggested by the American Congress of Rehabilitation Medicine [2]). The term total (or complete) LIS refers to a state of complete motor paralysis with no control over eye movements [3, 4].

Persons in the locked-in state require augmentative and alternative communication (AAC) to replace speech. These AAC systems can range from communication based on eye movements during face-to-face communication with caregivers to technical aids that allow for communication independent of the caregiver. While there are several high-tech communication aids available such as dynamic touch screens for persons who have residual muscular control [5], access options to AAC for persons in LIS are sparse. One of the most promising options is an eye-tracking based approach. A recent survey among 30 persons with advanced ALS using an eye-tracking computer system showed a high acceptance and average daily usage of 300 min of the device [6]. However, almost every fourth participant of the study (*n* = 7) reported a low daily utilization. Eyestrain and the inability to move the eyes sufficiently precise were the most frequent reasons reported for non-use. Another access option to AAC repeatedly proposed uses electrodes placed around the eyes of the user to record the electrooculogram (EOG) and thereby identify eye movements and/or blinks [7–9]. Similar to the eye tracker, this method relies on the users’ abilities to control their eye-muscles.

Brain-computer interfaces (BCIs) can provide a muscle-independent communication channel (for reviews, [10, 11]). A BCI based on event-related potentials (ERPs) in the electroencephalogram (EEG) was first proposed by Farwell and Donchin (1988 [12]) and ERPs are now the most widely applied control signals to enable communication with a BCI (for reviews see [13, 14]). Usually ERPs are elicited in so-called oddball paradigms. The users have the task of attending rare (odd) target stimuli in a series of frequent stimuli. These rare target stimuli elicit specific ERPs that can be classified and translated into computer commands. The most prominent among the elicited ERPs is the P300. It is a positive deflection in the EEG that occurs approximately 200–500 ms after the onset of a rare attended target stimulus with maximum amplitudes over central and parietal areas of the scalp [15]. For many years research focused on BCIs that apply visual stimulation to elicit ERPs. Most healthy users are able to control visual ERP-BCIs with high accuracies [16] and persons with severe paralysis were able to gain control over it [17–19]. Even control over complex applications such as a web browser, multimedia player and a painting application and long-term independent home use by ALS patients have been demonstrated [20–22]. Pasqualotto et al. [23] revealed a higher performance and usability for an eye-tracking system compared to a visual BCI with a group of persons with severe motor impairment. Nevertheless, there are situations in which a BCI might be advantageous. For instance, the participant with ALS of the long-term case study by Holz et al. [21] reported that it was less straining for her to make selection with the BCI, because unlike with her eye tracker, no eye blinks were required to make a selection. Because neither visual ERP-BCIs nor eye trackers can be controlled by persons with severe visual impairments and/or disability to control eye movements, e.g., persons in LIS, BCIs based on auditory and tactile stimulation were proposed in recent years (for a review see [24]).

Two types of auditory ERP-BCIs emerged. The first allows simple (binary) communication and is either based on attending target tones in a sequence of tones (sequential approach: e.g., [25–28]) or shifting attention to one of two auditory streams (streaming approach: [29–31]). Binary auditory BCIs are particularly suited for re-establishing simple communication with severely paralyzed persons since attentional and working-memory demands are low. The second type, multi-class BCIs, enable persons with long attention spans and good cognitive abilities to control spelling applications. For this, the number of tones to be differentiated in a sequential approach are increased ([32–38]).

The participant of the current study was in the locked-in state due to amyotrophic lateral sclerosis. At the time of the study, he did not use any AAC that would allow him to communicate independent of his caregivers and has not been using such technology previously. His family contacted us, because he had read about the possibility of using EOG and brain-computer interfaces as a method of communication in the study by Kaufmann et al. [8]. Although he could still communicate via eye movements, he wanted to test these methods as an alternative, since he had noticed a decline in his ability to control his eye movements. Hence, the aim of the study was to test a gaze independent BCI system that he could control without muscle activity and an EOG system as an alternative to his current method. We compared these systems to an eye tracking system. The advantages and disadvantages of each system are discussed, the prospects of using BCIs as assistive technology are reviewed and the need is emphasized for user-centered design in AAC in general and BCI development in particular.

## Methods

### Participant

At the time of the study, the Norwegian participant was 55 years old and has been in the locked-in state for 6 years. He was diagnosed with amyotrophic lateral sclerosis 9 years and 2 months prior to the study with first symptoms occurring 5 months prior to the diagnosis (muscle weakness in his legs). He was able to move slowly his eyes vertically and horizontally. Voluntary blinking was not possible. As the cause of an accident, he had lost hearing of his right ear. He was artificially ventilated (tracheostomy mechanical ventilation) and fed (percutaneous endoscopic gastrostomy). Full-time care was provided in his home, where the study was conducted.

### Conventional communication

To communicate with caregivers and family members the study participant relied on a letter board. The same groups of letters were printed on both sides of a cardboard frame. The caregiver or family member held the frame, facing the user. The user could then select letters with a two-step procedure. Via eye gaze he first selected a group of letters. The caregiver read out the letters of the selected group one at a time and the user indicated the letter he wanted to spell by slightly twitching his left eyebrow, when the desired letter was read. If control of eye movements was not possible due to fatigue, the first step was also done with partner assisted scanning (i.e., the caregiver pointed to the groups of letters one after the other and the user selected a group with a short twitch of his eye).

About 7 years prior to the study, the user had tried an eye tracking based system with Rolltalk communication software (Abilia AB, Sweden), but communication had worked better with the letter board described above.

### Procedure

On four consecutive days, the participant tested an EOG based system (3 sessions), an eye tracking based approach (1 session) and an auditory BCI (3 sessions) for communication. Since the participant was particularly interested in the EOG as an alternative communication channel, we started testing this system followed by the eye tracking and tested the BCI last. For all systems we optimized the parameters in a stepwise procedure to allow the participant to gain control over them. The procedure for each system is described below. Main parameters for each session are listed in Table 1. During the measurements, the participant sat in his wheelchair in a reclining position. He gave informed consent prior to participation (a signature stamp was used by his caregivers). The study was carried out in accordance with the guidelines of the Declaration of Helsinki.

**Table 1:** Parameters used and selections made during the measurements

	Session	Sequences	Possible choices	Selections
EOG				
Day 1				
*- control signal: looking to the left and back to the center*				
*- one electrode placed next to the outer canthus of the left eye*				
*- 1000 ms classification window*				
	*Training*	*10*	*2*	*2 runs*
	1.1	1	2	6
*- classification window changed to 2000 ms*				
	1.2	1	2	14
	*Training*	*- data from 1.2 used to train new classifer*		
	1.3	1	2	5
	1.4	1	5 (A, B, C, D, E)	5
*-voice recording by daughter*				
	1.5	1	5 (F, G, H, I, J)	15
*- added second electrode (next to canthus of right eye)*				
*- new control signal: looking to the right and back to the center*				
	1.6	1	5 (F, G, H, I, J)	10
Day 2				
*- stimulus presentation in alphabetical order*				
*- 1500 ms classification window*				
	*Training*	*3*	5 (F, G, H, I, J)	*1 run*
	2	1	5 (F, G, H, I, J)	19
Day 3				
	*Training*	*3*	5 (F, G, H, I, J)	*15*
*- two step procedure to select letter group and target letter*				
	3.1	1	5(25)letters A to Y	12
	3.2	2	5(25)letters A to Y	12
	3.3	2	5(25)letters A to Y	4
Eye Tracker				
Day 2				
	2	1	2	38
BCI				
Day 2				
	*Training*	*20*	*2*	*8 runs*
	2.1	10	2	3
	2.2	7	2	3
Day 3				
	*Training*	*20*	*2*	*6 runs*
	3.1	20	2	2
	3.2	7	2	3
	3.3	10	2	6
Day 4				
*- classifier from day 3 applied*				
	4.1	20	2	4
*- data from 4.1 added to data from day 3 to train new classifier*				
	4.2	10	2	4

### Data acquisition

Stimulus presentation, data processing and storage were controlled by the BCI2000 software framework [39]. EEG data during BCI and EOG use was amplified with a g.USBamp (g.tec, Austria) with a sampling rate of 256 Hz, a bandpass filter from 1 to 30 Hz and a notch filter around 50 Hz. The EyeX eyetracker was connected to BCI2000 using the software development kit provided with the hardware by Tobii Technology. Recordings were made with a Hewlett-Packard ProBook 6460b with a dual-core CPU, 4GB of RAM and a 64-bit Windows 7.

### EOG

The eye movement that the participant used to communicate during the partner scanning approach (twitching his left eyebrow) was not strong enough to be registered with an electrode placed above his left eyebrow. Thus, we asked the participant to perform another eye movement that he could control reliably and that was not too strenuous. The participant chose looking to the left and back to the center as control signal for the EOG. To record this movement we placed one electrode next to the outer canthus of his left eye. Stepwise linear discriminant analysis (SWLDA, [40]) was applied to determine features and feature weights of the EOG data acquired during the training runs that were subsequently used for online classification during the feedback runs. During both the training and the feedback runs we asked the participant to respond with an eye movement to a predefined target letter. Voice recordings of the letters “A” and “B” were played in random order via the built in speakers of the notebook. Presentation of these two letters constituted one sequence. Stimulus duration was set to 1 s and the interstimulus interval (ISI) to 2.5 s, hence stimulus onset asynchrony (SOA) was 3.5 s. During the feedback runs, the selected letter, “A” or “B”, was presented after a short signal tone. The pause between sequences was set to 5 s, thus 12 s were needed for one selection with this two choice paradigm. Later during that session the participant decided to switch to “looking to the right and back to the center” as control signal for the EOG. Therefore, we placed an additional electrode next to the outer canthus of his right eye and used the differential activity of both electrodes to classify his eye movements. Because eye movements of the participant were slow, we changed the time window for classification from originally 1000 to 2000 ms during the process of testing (see Table 1 for an overview of the applied parameters).

To increase the number of possible selections to 5 we presented the letters A, B, C, D and E. To increase the discriminability of the letter recordings (the letters D and E sounded similar), we later asked the daughter of the participant to record the letters F, G, H, I and J in Norwegian. These letters were played in random order and the participant was instructed to respond with eye movements to the target letter. On day 2, we facilitated the task by playing the letters in alphabetical order. On both days, the feedback consisted of the chosen letter that was played after a short signal tone. Selection of one letter took 22.5 s with one sequence. On day three we asked the participant to spell few words with a two-step procedure. First, we asked the participant to select a group of letters (A-E, F-J, K-O, P-T or U-Y) and in the second step the target letter within the chosen group. The groups of letters and letters within the chosen group were each coded by the spoken numbers 1 to 5. Therefore, he had to respond to the number 1 and after a short break of 7.5 s to the number 3 to select the letter “C”. To facilitate the task for the participant, the assignment of groups of letters and numbers (e.g., during the first step A, B, C, D, E = 1; during the second step A = 1) were presented to the participant on a sheet of paper. Feedback about the chosen group and letter was provided acoustically after every step. Selection for one step took 25 s, hence the time needed to spell one letter was 50 s with this paradigm.

### Eye tracking

**Fig. 1:**
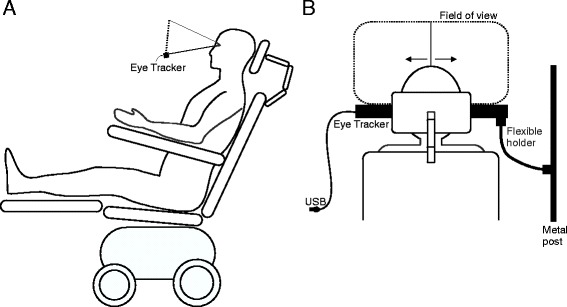
Schematic figure depicting the position of the eye tracker in front of the user. Lateral view (**a**) and rear view (**b**). The user’s fixation point could be determined in the area above the eye tracker. The fixation point was determined as being either in the left or right half of this area and thus, the user could make a binary choice

Eye movements were recorded with a Tobii EyeX Dev Kit (Tobii Technology, Sweden) that is based on the principle of corneal reflection tracking. The eye tracker was attached to a metal post using a flexible holder and positioned in front of the user such that the eyes could be recognized by the system (see Fig. 1). The area above the eye tracker (corresponding to a screen size of 1680 × 1050 pixels) constituted the area in which the gaze point could be determined. We ensured that this area was within the participant’s field of view. To calibrate the system, we asked the participant to follow the predefined movements of a pen that we held in his field of view. To allow for a comparison with the EOG, we chose looking to the left or right as control signal. Similar to the procedure for the EOG, the letters “A” and “B” were played over speakers (one sequence). Before each run, the user was asked to respond to one of the two. If “A” was the designated target, the user was asked to look to the left and for “B” to the right. The gaze point was determined at the end of each sequence. During free runs we asked the user to respond to either of the two stimuli and asked him afterwards which selections he had aimed for. Stimulus duration was set to 1 s and the inter-stimulus interval to 1.5 s. Between sequences of letters there was a pause of 3 s in which the user was instructed to look straight ahead and during which he received acoustic feedback. A voice recording saying “left” or “right” was played depending on the determined position of the gaze point (in the left or right half of the field of view). Including the time for feedback, 8 s were needed for one selection.

### Auditory BCI

During BCI use, the EEG was recorded with 16 active Ag/AgCl electrodes mounted in an elastic fabric cap and positioned at FC3, FCz, FC4, C3, Cz, C4, CP5, CPz, CP6, P3, Pz, P4, PO5, Poz, PO6 and Oz according to the modified 10–20 system of the American Electroencephalographic Society [41]. A ground electrode was positioned at AFz and the data was referenced to an electrode clipped to the right earlobe. The EEG was amplified with a g.USBamp (g.tec, Austria) and sampled at 256 Hz. A notch filter around 50 Hz and a bandpass filter between 0.1 and 30 Hz were applied. Auditory stimuli were presented over circumaural headphones (Sennheiser HD 280 pro, Germany).

The task consisted of a three stimulus oddball paradigm as suggested for binary BCI communication by Halder et al. [26]. Three different tones were presented in random order: A high pitched target tone with a frequency of 1000 Hz, a low pitched target tone of 100 Hz and a standard tone which consisted of pink noise. One sequence consisted of three standard stimuli and the two target tones. All stimuli had a duration of 80 ms and the stimulus onset asynchrony was 1000 ms. The participant was instructed which target tone to attend to before each run. He was asked to focus on the appearance of that tone and silently count whenever it sounded and ignore all other tones. To acquire data to train the classifier, 20 sequences were played per training run. Stepwise linear discriminant analysis [40] was applied to determine features and feature weights for online selections with the BCI. During these online runs, the number of sequences was reduced and the user received feedback according to the classifier results. Hence, the time needed for one selection depended on the number of sequences (e.g., with 10 sequences: 50 s plus 2 s for feedback).

### Questionnaire

At the end of testing, after the fourth session, we presented a summary of the achieved performance and the general advantages and disadvantages for each of the tested systems to the participant (similar to the ones summarized in Table 2). To gather his feedback, we then asked him the same set of questions for all three systems. The questions are listed below. We started with the questions about the EOG and ended with the set of questions for the BCI. The participant answered all questions with his conventional communication method (partner scanning approach with eye movements).

**Table 2:** Characteristics of the tested systems

	EOG	Eye Tracker	BCI
Allows communication independent of the caregiver	yes	yes	yes
Enables muscle-independent communication	no	no	yes
Speed of communication	medium	fastest	slowest
Commercially available			
• Hardware	yes	yes	yes
• AT software and support	no	yes	no
Costs	medium	lowest	highest
Burden on the caregiver	medium	lowest	highest
Applications	communication	communication	communication (binary)

- How difficult/easy was it for you to control the EOG/eye tracking/EEG(BCI) based system, on a scale from 0 to 10 (if 0 = very difficult and 10 = very easy)?
- How tiring was using the EOG/eye tracker/BCI for you, on a scale of 0 to 10 (if 0 = not tiring at all and 10 = extremely tiring)
- How long (hours/minutes) do you think you would be able to use it before you would need a longer break?
- Given that your current method of communication still works, would you consider using EOG/eye tracking/BCI as an additional communication method?
  If no, which are the obstacles of use?
  If yes, what would be the most important improvement?
- Would you consider using the EOG/eye tracking/EEG(BCI) based system if your current AT system was no longer working?
  If no, which are the obstacles of use?
  If yes, what would be the most important improvement?

## Results

### Performance

**Fig. 2:**
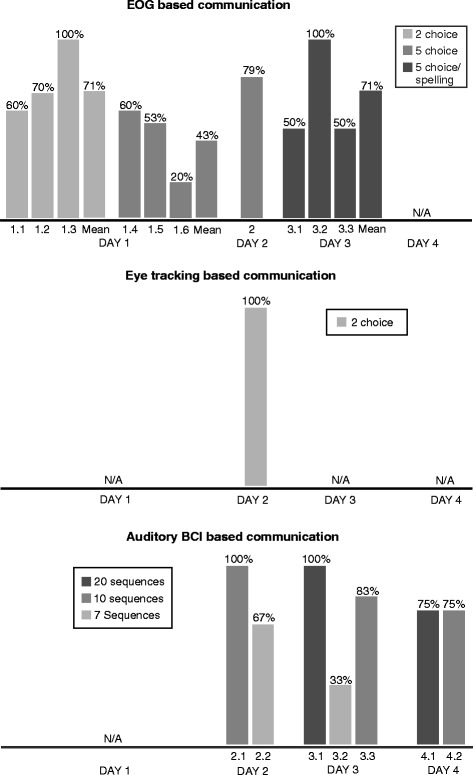
Online selection accuracies for all tested systems and sessions. The mean accuracies for the EOG based system were calculated by weighting the depicted accuracies by the number of selections made. Starting from day 2, stimuli were presented in alphabetical order for the EOG based system and in session 3.2 and 3.3 two sequences were used instead of one. Please refer to Table 1 for a detailed listing of the applied parameters and selections made in each session

The participant was able to control the EOG, eye tracking and BCI based system. Figure 2 shows the performance comparison for all tested systems across all sessions.

With the EOG based system the user reached above 70 % accuracy with the two choice paradigm on the first day, but had difficulties with five selections on the same day. When we presented the 5 letters in alphabetical order on day two, he reached an accuracy of 79 %. On day 3, we introduced the two step procedure that theoretically allowed him to choose any letter of the latin alphabet except the Z. He reached 100 % accuracy in session 3.2 in which we asked him to spell two 3 letter words (12 selections in total), but two sequences (stimulus repetitions) were needed. In session 3.3 the user tried to spell a word of his choice, but stopped after the third attempt (fourth selection), because he could not concentrate on the target sounds necessary to select the desired letters (unable to recall the position in the spelling tree).

With the eye tracking based system all selections were classified correctly. However, placing the eye tracker in front of the user in a way that the system could recognize the reflections of the light source on his cornea and in his pupil was difficult. Set-up and calibration took about 20 min. The user had difficulties looking in a particular direction; therefore we facilitated the task for the first 12 selections, by holding a pen above the eye tracker within the designated target half to help him fixate. For the remaining selections, in which the user could choose the side he wanted to look to, we asked him to only make small eye movements to either of the sides. The system still identified all intended selections correctly.

With the auditory BCI, the study participant reached accuracies above 70 % on all three days of testing. To reach this threshold, 10 or 20 sequences were needed during online selections. Exemplary EOG and EEG traces are provided as Additional files 1 and 2.

### User feedback

**Fig. 3:**
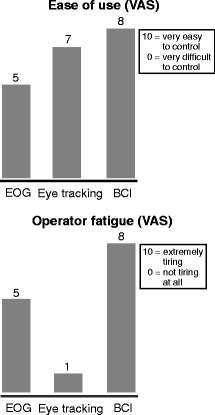
User ratings (VAS) of fatigue and ease of use for the tested systems

Figure 3 depicts a comparison of the user ratings regarding the ease of use and the operator fatigue for all systems. The user indicated that he would not consider any of the tested systems as an additional method of communication, although he estimated that he could use the EOG for about 2 h, the eye tracker for 4 h and the BCI for about 1 h before he would need a longer break. The obstacles of use for the EOG system were the strong eye movements required for the system to work and he stated that it was unlikely that the system would be able to detect movements that could not also be detected by the caregiver. Although the eye tracking system would allow him to communicate independent of the caregiver, he expressed a clear preference for the partner scanning based approach. The only system that he would consider using if he was no longer able to control his current method, was the BCI. He did not think that the other systems could help him in that case. He considered the ease of use of the BCI as the highest among the tested systems, but also rated it as the most tiring.

## Discussion

The study demonstrated that a person in the locked-in state can gain control over an EOG based system, an eye tracker and an auditory BCI for binary communication with satisfactory accuracies (>70 % correct). Nevertheless, the participant expressed a preference for his low-tech method of communication over the tested systems and did not consider using them as an additional method. The only system that he would consider as a communication aid is the BCI, in case the partner assisted scanning approach (with a letter board based on eye movements) was no longer working.

The focus of this case study was on testing possible input signals that could be implemented to control AAC devices, rather than providing ready-to-use communication applications. Due to the study design with only one participant, it is important to stress that generalization to the general population of persons in LIS is not possible. The iterative process of tailoring the different technologies to the participant’s needs prevented us from comparing them in terms of assistive efficacy. Therefore, we will discuss in detail the results obtained for the participant and only briefly address the potential of each system as AAC device with references to other studies.

An overview of the main features of the tested systems is provided in Table 2. In the following, we will discuss the results obtained with each system and considerations for future work.

### Electrooculogram

The participant rated the ease of use of the EOG lowest. The main reason was the relatively strong horizontal eye movements required that were strenuous for the participant. While it was therefore not an option for the participant as an input signal for AAC, it could be a good option for some users. For instance, Kaufmann et al. [8] showed that lifts of the eyelid of their study participant with LIS could reliably be classified and used for spelling of words after a short calibration phase.

### Eye tracking

The participant of the current study rated the eye tracker as the least tiring of the tested systems. A similar result was obtained by Pasqualotto et al. [23], who could show that the workload of an eye tracker was lower than that of a P300 BCI. Although an eye tracking based system would allow for communication independent of the caregivers or even control of technical devices (e.g., a TV or radio), the participant of our study did not consider it as an additional communication method. The participant had full-time care and with most caregivers and family members, who we met during the study, he had achieved a high speed of communication. This was mostly due to the familiarity of the caregivers and the participant. Hence, the communication partners could suggest letters or words based on their personal knowledge about the participant and based on the context or topic of the conversation. They could also react flexibly to his ability to move the eyes and adapt the partner scanning approach accordingly. This level of flexibility and efficiency would be difficult to reach with an eye-tracking based system. Another reason for his preference of the low-tech method may have been that during the partner assisted communication method there is a direct interaction between the user and the interlocutor.

Despite the demands on the communication partners, the spontaneous face-to-face conversation mode was reported by caregivers to be the most frequent with persons with ALS in a study by Fried-Oken et al. [42]. Similarly, in a study by Spataro et al. [6] half of the study participants, who had an eye tracking system available, communicated regularly with a letter board. Further, there is evidence that these low-tech methods are predominant for persons with most severe levels of disability (e.g., persons in the locked-in state) [5, 6, 43]. Nevertheless*,* for those persons with no residual motor control except eye movements, it is often the only (commercial) method to gain access to AAC. Studies including persons with late-stage ALS could show an improvement in the quality of life for persons using an eye tracking system, a high satisfaction with the system for most participants and a reduced burden on caregivers [6, 44–46]. Shortcomings frequently reported are difficulties operating under a range of changing light and postural conditions, determining the optimal dwell time and technical support required [5, 43, 47, 48].

### Brain-computer interfaces

In this study we tested an auditory ERP-BCI with simple to understand instructions (“please listen to the high/low pitched tone”) that would allow for binary communication [26]. The participant achieved a satisfactory level of control. He rated the ease of use as the highest of the tested systems, but also described it as the most tiring. These ratings suggest that the ease of use was rated as high since no muscle movements were required, but that the attentional demands were substantially higher for the BCI compared to the other systems. Due to the deafness of his right ear, we could not test a streaming approach as suggested by Hill & Schölkopf [29, 30] that requires dichotic listening. For a person with intact hearing, a streaming approach could substantially speed up the selection rate. Another improvement could be to replace the beep sounds by more natural sounds, such as recordings of yes or no [28, 31].

For persons with long attention spans and sufficient cognitive abilities, auditory multi-class BCIs could provide spelling solutions (e.g., [33–38]). With training, a satisfactory level of control could be achieved [49, 50]. Tactile ERP-BCIs could be an option for persons who are unable to control auditory BCIs [8, 24].

In case all voluntary muscular activity is lost, i.e., in the complete locked-in state, communication based on recordings of brain-activity might still be possible. Brain-computer interfaces enabled severely paralyzed persons, even users in the locked-in state, to communicate [17, 18, 51, 52]. However, only few reports on communication attempts with persons in the complete locked-in state exist [53, 54]; see [17] for an overview) and only one reported significantly above chancel level performance [55].

### Considerations for future work

Brain-computer interfaces could prove valuable for participants in situations when the preferred method is not operable due to muscular fatigue among others. For these situations, a robust and simple to operate BCI is needed, particularly for those users, who usually rely on low-tech partner assisted communication such as the participant of this study. For persons, who use more advanced AT such as an eye tracker, a complementary or hybrid approach could ease the transition to a purely BCI based AT if control over all muscles is lost (i.e., in CLIS caused by a neurodegenerative disease). Recently hybrid BCIs (hBCIs) were proposed that consist of a combination of one or more conventional AT input devices or biosignals and at least one BCI channel (for a review of the state-of-the-art see [56]). Although case studies demonstrated the feasibility of long-term independent home use of BCIs, [21, 22, 57], usability has to be improved before BCIs be considered as assistive technology for a larger group of persons. To achieve this goal, it is important to engage the potential end-users during all steps of the development process from the definition of user requirements to the implementation and the iterative testing of prototypes [58]. Measures to operationalize usability for the evaluation of BCIs were proposed by [21, 59, 60] among others.

## Conclusions

We demonstrated that a person in the locked-in state was able to gain control over an EOG, an eye tracker and an auditory brain-computer interface. Due to the end user’s and his caregivers’ proficiency in using a low-tech communication method based on residual eye movements, he did not consider using the tested systems as an access method to AAC. He would consider using a BCI once control over his eye muscles will be no longer possible.

BCIs can extend the range of available access methods and the combination with existing assistive technology should be considered. A user-centered design approach in the development of these systems will increase the likelihood that they will be used as assistive technology in daily life. For persons with severe paralysis, who could benefit from a BCI (immediately or in the future), solutions tailored to the users’ individual needs are required and thus, the engagement of targeted end-users in all steps of the development process is needed as requested by the user-centered design [58].

## Additional files

Additional file 1: Figure S1. Average EOG traces for the training run on day 3. The task of the participant consisted of looking to the right and back to the center in response to the target letter. (PDF 18 kb) Additional file 2: Figure S2. Averaged ERP waveforms at Cz; and scalp plot of differential ERP activity (targets minus non-targets) at the peak latency of the P300 for session 2.1. (PDF 88 kb)
