# Supplementary figures and images for: Comparison of eye tracking, electrooculography and an auditory brain-computer interface for binary communication: a case study with a participant in the locked-in state
Source: J Neuroeng Rehabil. 2015 Sep 4;12:76. doi: 10.1186/s12984-015-0071-z (PMC4560087; doi:10.1186/s12984-015-0071-z)

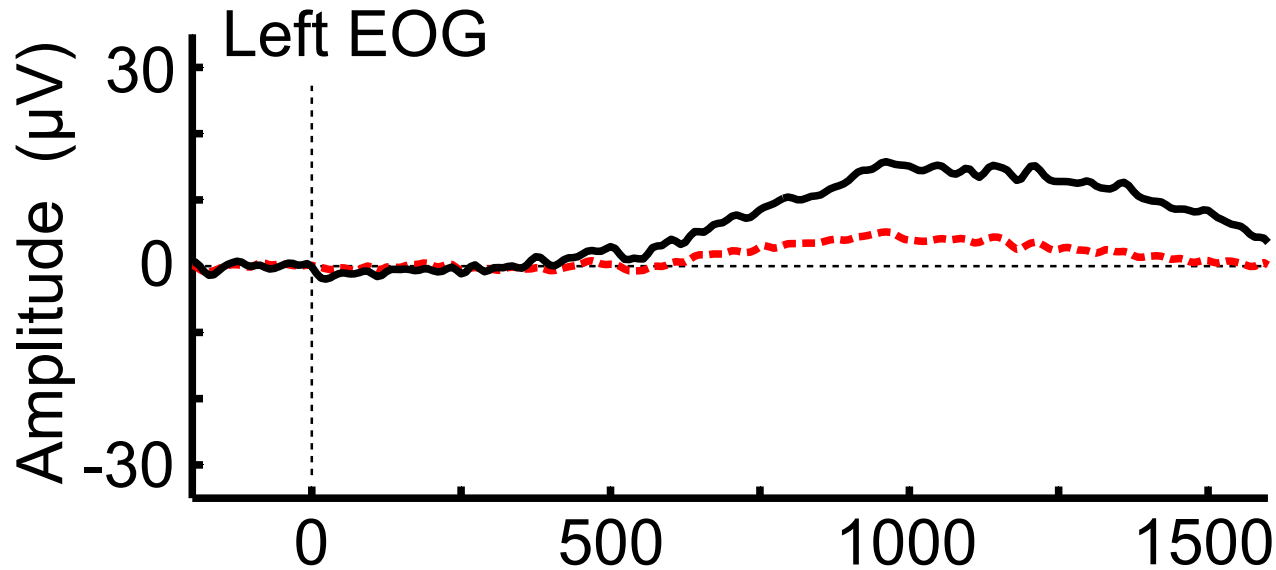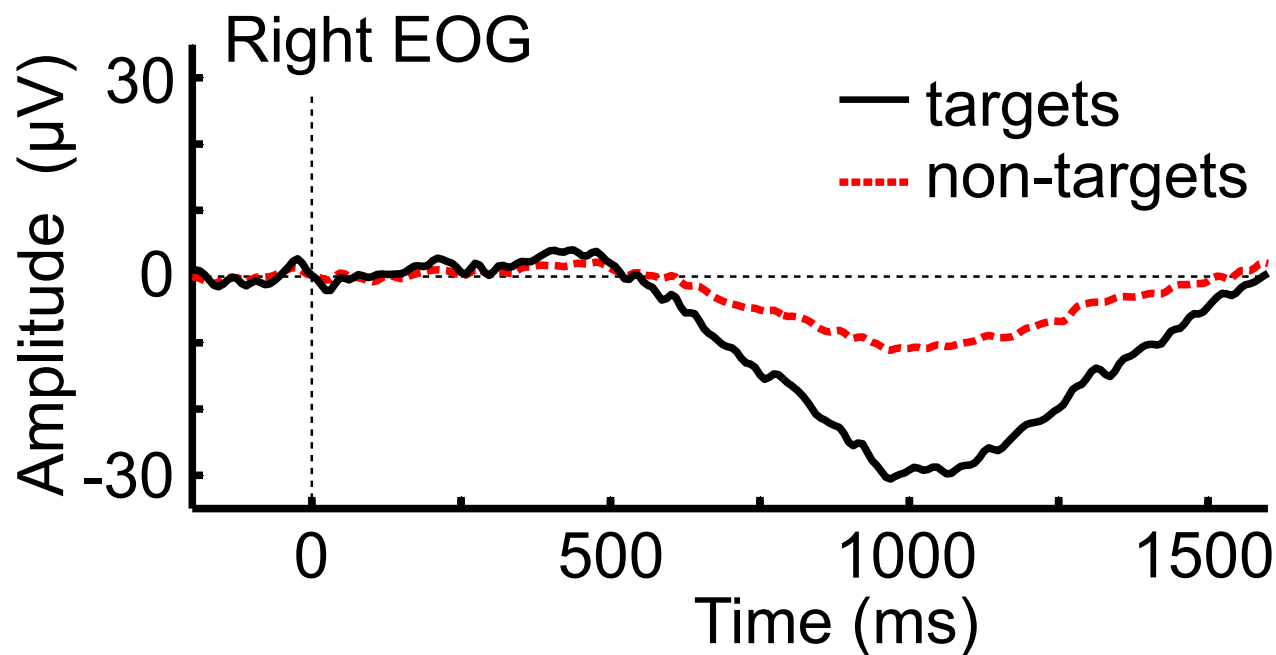

Supplement: Additional file 1: Figure S1. — Average EOG traces for the training run on day 3. The task of the participant consisted of looking to the right and back to the center in response to the target letter. (PDF 18 kb) [file 12984_2015_71_MOESM1_ESM.pdf]

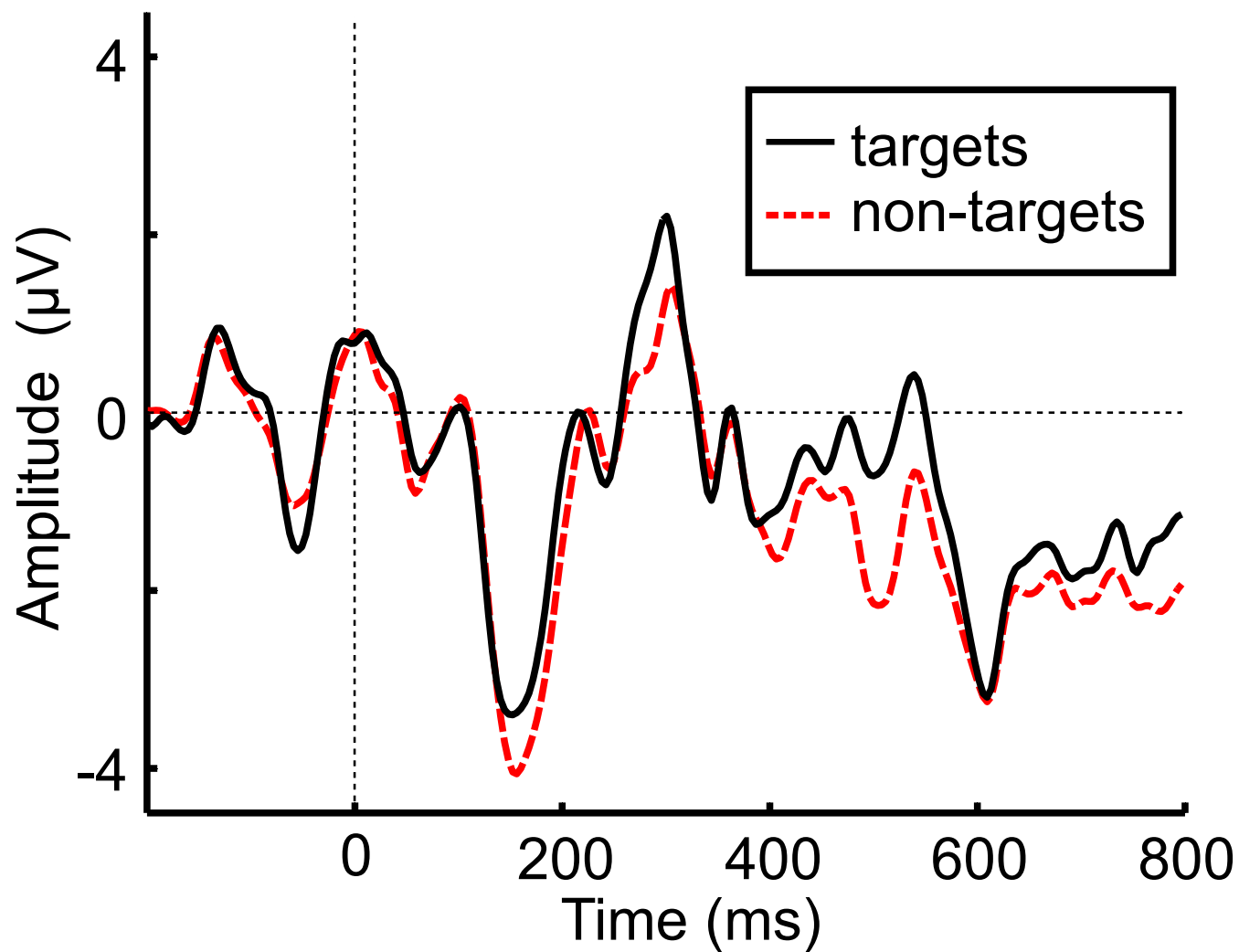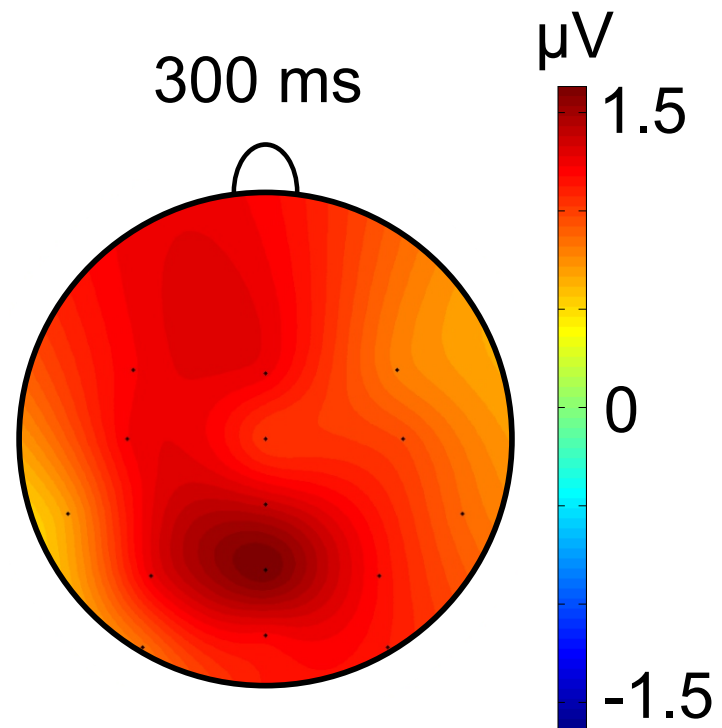

Supplement: Additional file 2: Figure S2. — Averaged ERP waveforms at Cz; and scalp plot of differential ERP activity (targets minus non-targets) at the peak latency of the P300 for session 2.1. (PDF 88 kb) [file 12984_2015_71_MOESM2_ESM.pdf]
